# Branching projections of ventrolateral reticular neurons to the medial preoptic area and lumbo-sacral spinal cord

**DOI:** 10.1186/1744-9081-1-17

**Published:** 2005-10-07

**Authors:** Antonella Russo, Rosalia Pellitteri, Rosa Romeo, Stefania Stanzani, André Jean

**Affiliations:** 1 Department of Physiological Sciences, University of Catania, Catania, Italy; 2 Institute of Neurological Science, Research National Council, Catania, Italy; 3 Department of Anatomy, Diagnostic Pathology, Phorens Medicine, Hygiene and Public Health, University of Catania, Catania, Italy; 4 Laboratoire de Physiologie Neurovégétative, UMR 6153-CNRS 1147-INRA, Université Aix-Marseille III, Faculté des Sciences St. Jerôme, Marseille, France

**Keywords:** ventrolateral reticular nucleus (RVL), retrograde fluorescent tracer, medial preoptic area, spinal cord, rat.

## Abstract

Different findings indicate that rostral ventrolateral reticular nucleus (RVL) is neuronal substrate of integration and regulation of the cardiovascular functions. Some efferent RVL neurons project to the thoraco-lumbar spinal cord and excite preganglionic sympathetic neurons, to the spinal phrenic motor neurons involved in inspiratory function and increase the activity of vasoconstrictor fibres innervating blood vessels in the skin and skeletal muscle. Our study was aimed at revealing presence of neurons within RVL supplying branching collateral input to the medial preoptic area (MPA) and to the lumbo-sacral spinal cord (SC-L) in the rat. All animal experiments were carried out in accordance with current institutional guidelines for the care and use of experimental animals. We have employed double fluorescent-labelling procedure: the projections were defined by injections of two retrograde tracers: Rhodamine Labelled Bead (RBL) and Fluoro Gold (FG) in the MPA and SC-L, respectively. Our results showed the presence of few single FG neurons and single RBL neurons in the RVL. The size of FG-neurons and RBL-neurons was medium (25–30 μm) and large (50 μm).

Few double-projecting neurons were distributed in the middle third of RVL nucleus, their size was 30–40 μm. The results demonstrate that pools of neurons in the RVL have collateral projections to the MPA and SC-L and they are involved in ascending and descending pathway. These data suggest that these neurons could play a role in maintaining activity of central and peripheral blood flow.

## Introduction

Several findings indicate that rostral ventrolateral reticular medulla (RVL) is the fundamental neuronal substrate of the regulation of circulation and cardiovascular functions, that mediates vasomotor reflexes [1,2]. Different methods permitted to identify afferent projections to RVL, from the hypothalamic paraventricular nucleus [3], the lateral hypothalamic area [4], the dorsal raphe nucleus [5] and from other regions [4]. Some efferent RVL neurons project to the intermediolateral cell column of the thoraco-lumbar spinal cord and excite preganglionic sympathetic neurons [6-8], spinal phrenic motor neurons involved in inspiratory function [9] and increase the activity of vasoconstrictor fibres innervating blood vessels in the skin and skeletal muscle [10]. Ascending RVL efferent projections convey informations from RVL to diencephalic nuclei [4,11] and collateralized fibres were found in RVL, after injections within the cerebellar fastigial nucleus and the superior colliculus [12]. In fact, electrical stimulation of RVL in rats elicits an excitatory response on the renal sympathetic nerve activity [7,13]. The MPA is also involved in controlling of numerous functions: neuroendocrine, sexual, maternal behaviour and other activities [14-16]. The aim of the present study is to demonstrate the existence of direct projections from RVL to the MPA and, *via *collaterals, the existence of direct projections to MPA and SC-L. In addition, previous work has shown that RVL adrenergic (C1) and non-adrenergic neurons were found to participate in these projections [17-19]. The brainstem neurons may be involved in simultaneous transmission of autonomic-related signals, in fact catecholaminergic and non-catecholaminergic neurons were found to provide branching collaterals to the central nucleus of the amygdala and to the hypothalamic paraventricular nucleus [11].

In the present study we have used two retrograde tracers to determine the distribution of RVL neurons that project *via *collaterals to the MPA and SC-L.

## Results

**Figure 1:**
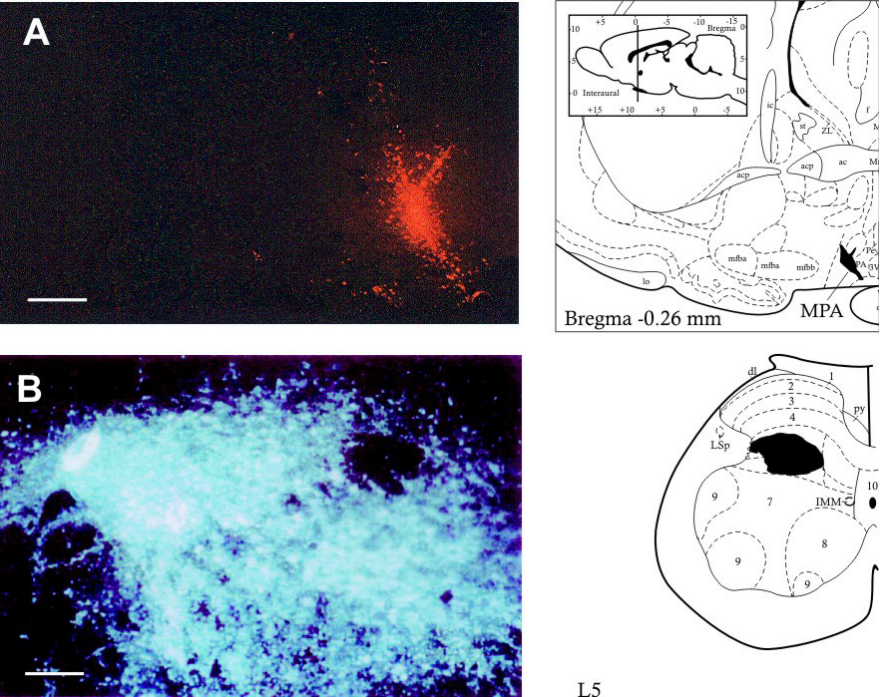
Microphotographs show injection zones: in (A) RLB injection site and drawing in MPA (black area); Scale bar: 400 μm. In (B) FG injection site and drawing in SC-L (black area). Scale bar: 90 μm.

Only those cases (8 rats) where microscopic analysis of the injection sites revealed that the tracer deposits were correctly positioned were included in the study. Fig. 1 shows the injection sites of the retrograde tracers RLB in the MPA (A) and FG in the SC-L (B). The results showed a large number of retrogradely-labelled neurons in the whole ipsi- and contralateral RVL (stereotaxic planes: -11.60/-11.96). FG-labelled neurons from the ipsilateral and contralateral RVL were numerous (39.42 ± 3.3); moreover, these neurons ranged in size from medium (20–30 μm) to large (50 μm) and were rather sparse within RVL. RLB-labelled neurons were mostly packed within the boundaries of the field examined, consistently of medium size (20–35 μm) and uniformly distributed in ipsi- and contralateral RVL (38.2 ± 2.85). The fluorescence microscopy revealed a substantial number of RVL double-labelled neurons (18.6 ± 3.13), evidencing the presence of collateralization to the MPA and SC-L. These neurons generally were of medium to large size (30–40 μm) and were localized in the middle third of RVL.

**Figure 2:**
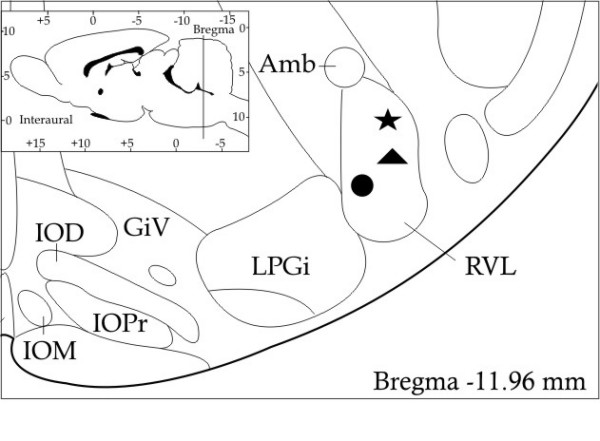
Schematic drawing atlas of frontal brain section including RVL region: circle indicates FG labelled neurons; triangle indicates RLB labelled neurons; star indicates FG-RLB labelled neurons.

Fig. 2 shows schematic drawing of frontal brain section including RVL region with different symbol (circle, triangle and star) indicating the labelled neurons presence.

**Figure 3:**
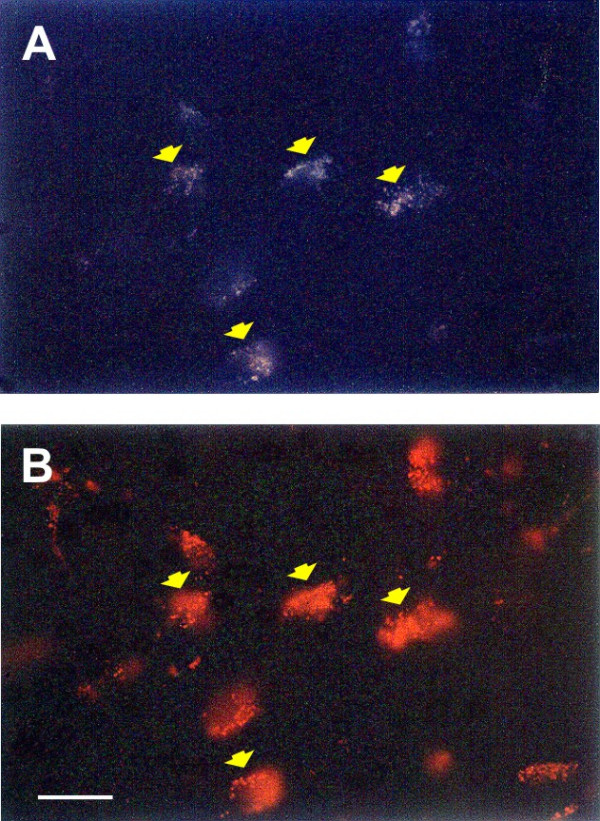
Microphotographs of double-labelled neurons FG-RLB in reticular ventrolateral nucleus (RVL). (A) cells stained positively to FG (excitation wavelength 330 nm); (B) the same cells stained positively to RLB (excitation wavelength 560 nm) indicating the existence of a collateral axon. Scale bar: 50 μm.

A relatively low number of FG/RLB (4.6 ± 1.49 neurons) double-labelled neurons (30–40 μm) were scattered mainly at the ipsilateral RVL level; an example is showed in Fig. 3.

## Discussion

The present study provides direct evidence, based on retrograde tracing technique, that: 1) RVL single neurons directly project to the medial preoptic area; 2) RVL single neurons directly project to spinal cord, confirming previous results [4]; 3) RVL neurons supply, *via *collaterals, branching inputs to the MPA and SC-L. Sympatho-excitatory neurons of the RVL, like in rostral ventromedial medulla [20] and in caudal ventrolateral medulla [21], are important for the maintenance of tonic levels of arterial pressure they are intrinsic pacemaker activity and discharge continuously [2]. In conclusion, after the injections of FG and RLB, we show a cluster of branching RVL neurons in the rat brain. The fact that RVL contains neurons with a collateral fibers to both the spinal cord and to hypothalamic MPA, suggests that these neurons might play a role in maintaining activity of central and peripheral blood flow simultaneously.

## Materials and methods

All experiments were carried out in accordance with current institutional guidelines for the care and use of experimental animals. Experiments were performed on 10 adult male Wistar rats weighing 250–300 g (Morini, Italy), maintained under controlled conditions of room temperature (23 ± 1°C) and lighting (lights on 07:00 – 19:00 h); laboratory chow diet and water were available *ad libitum*; the *in vivo *experimental procedure was performed during daytime (10:00 – 13:00 a.m.).

Animals were anaesthetized with chloral hydrate (400 mg/Kg, i.p.). Two fluorescent tracers were injected to the same rat: Fluoro Gold (FG) was injected into the lumbo-sacral spinal cord on one side, Rhodamine Labeled Bead (RLB) into the medial preoptic area of the same side. Rats were placed in a Kopf stereotaxic frame and injected with 0.08 μl of undiluted RLB in the MPA, at the following coordinates (0.30; L = 0.5; V = -8.5) [22]; freshly dissolved FG (0.15 μl at 6% in saline) was injected into the SC-l. Both tracers were pressure-injected at a rate of 50 nl/min. using 1 μl Hamilton microsyringes.

Seven days after the injections, the animals were reanaesthetized and perfused through the ascending aorta with saline (60 ml), followed by ice-cold 4% paraformaldehyde phosphate buffer (300 ml; pH 7.4). The brains were removed, immersed in the same fixative for 3–4 h and cryoprotected overnight in phosphate-buffered with 20% sucrose solution.

Coronal sections (40 μm) were cut on a cryostat (Reichert), mounted on slides and observed under fluorescent microscope (Polyvar Reichert) for identification of injection zones. The sections were air-dried, mounted and observed with a Reichert fluorescence microscope equipped with filter combinations revealing red (RLB), yellow (FG). For each animal, three non-adjacent sections were evaluated and the labelled cells plotted onto schematic drawings of the RVL region level (stereotaxic planes: -11.60 / -11.96) [5]. Thus, cell numbers were expressed as the average number/section calculated from these three sections.

## Competing interests

The author(s) declare that they have no competing interests.

## Authors' contributions

AR, RP, RR, SS and AJ jointly conceived and executed this study and helped draft the manuscript. All authors read and approved the final manuscript.
